# Maximum acceptable frequency of lift for combined manual material handling task in Malaysia

**DOI:** 10.1371/journal.pone.0216918

**Published:** 2019-05-29

**Authors:** Mirta Widia, Siti Zawiah Md. Dawal, Nukman Yusoff

**Affiliations:** 1 Occupational Safety and Health Program, Faculty of Engineering Technology, Universiti Malaysia Pahang, Gambang, Pahang, Malaysia; 2 Department of Mechanical Engineering, Faculty of Engineering, University of Malaya, Kuala Lumpur, Malaysia; Instituto Politecnico de Viana do Castelo, PORTUGAL

## Abstract

**Background:**

Extensive studies have been carried out over the years to determine the maximum acceptable weight that a worker is capable of lifting in a given situation among Occidental populations across Europe and US. Nonetheless, studies that place emphasis on using lifting frequency as the quantifying task parameter, especially in developing countries such as Malaysia, appear to be in scarcity. Hence, this study determined the maximum acceptable frequency of lift (MAFL) for combined manual material handling (MMH) tasks amongst Malaysian males.

**Method:**

Two lifting loads were considered in this study: 1 kg and 5 kg. Each subject adjusted his frequency of lifting using a psychophysical approach. The subjects were instructed to perform combined MMH task as fast as they could over a period of 45 minutes without exhausting themselves or becoming overheated. The physiological response energy expenditure was recorded during the experimental sessions. The ratings of perceived exertion (RPE) for four body parts (forearms, upper arm, lower back and entire body) were recorded after the subjects had completed the instructed task.

**Results:**

The mean frequencies of the MMH task had been 6.8 and 5.5 cycles/minute for lifting load of 1 and 5 kg, respectively, while the mean energy expenditure values were 4.16 and 5.62 kcal/min for 1 and 5 kg load, respectively. These displayed a significant difference in the Maximum Acceptable Frequency of Lift (MAFL) between the two loads, energy expenditure and RPE (*p* < 0.05) whereby the subjects appeared to work harder physiologically for heavier load.

**Conclusion:**

It can be concluded that it is significant to assess physiological response and RPE in determining the maximum acceptable lifting frequency at varied levels of load weight. The findings retrieved in this study can aid in designing tasks that do not exceed the capacity of workers in order to minimise the risk of WRMSDs.

## Introduction

Work-related musculoskeletal disorders (WRMSDs) are bound to become the leading cause of disabilities with serious implications for both society and public health by 2020 [1]. Manual material handling (MMH) tasks are an important contributor of lower back problems and other WRMSDs [2–4]. WRMSDs due to MMH tasks have long been reckoned as one of the main occupational injuries that has affected the quality of life amidst the industrial working population in the US and other nations [5, 6]. In Taiwan, approximately 59% of Taiwanese workers were injured from MMH tasks [7]. The number of patients with lower back problems recorded in the South Korean industry escalated by an astounding figure (246%) from 2002 to 2003 –a span of only a year [8]. Preventing WRMSDs has been now considered as a national priority in a number of nations including Malaysia [9, 10]. A report from the Malaysian Department of Occupational Safety and Health (DOSH) revealed that the highest number of industrial accidents was contributed by the manufacturing sector with a total of 1649 cases [11]. The most affected location of injury due to industrial accidents was the upper limb region with 30,356 cases (44.6%), followed by the lower limb region with 13,475 cases (19.8%) [12]. The number of MSD cases increased drastically from 15 cases in year 2006 to 675 cases in 2014, which is roughly 44 times the original number of cases within a span of eight years [13]. The compensation cost paid due to such injury had been higher when compared to other industrial diseases [14]. The Malaysian Government had even paid RM 1.4 billion for industrial workers’ compensation claims through SOCSO in the financial year of 2010.

A large and growing body of literature has developed some guidelines and has determined the maximum acceptable weight of lift (MAWL) for all those involved in MMH tasks based on psychophysical, physiological and biomechanical approaches as measures to prevent WRMSDs. The National Institute for Occupational Safety and Health (NIOSH) work practices guide and lifting equations [15] were developed in accordance to the following guidelines: (1) American Conference of Governmental Industrial Hygienists (ACGIH), (2) Threshold Limit Values (TLVs) for Lifting and (3) Snook and Ciriello Psychophysical Tables. To date, physiological approaches on the energy expenditure is the most widely accepted measure for physiological response to repetitive handling since it is directly proportional to the workload at steady-state conditions [16–21].

Most studies have determined the MAWL for individual task, such as symmetric lifting task [6, 22–24] and asymmetric lifting task [6, 7, 25]. Hidalgo and Genaidy [26] introduced a comprehensive lifting model (CLM) for evaluating and designing of manual tasks. Karwowski [27] determined the ‘safe’ lifting capacities on the maximum safe weight of lift (MSWL). He found that the MSWL values were significantly lower than those of MAWL. He suggested that human judgement of load heaviness should be modelled using the cognitive engineering approach [28]. Nonetheless, only a handful of studies have looked into MAWL or lifting capacities limit of combined two or more MMH tasks, such as lifting, carrying and lowering loads. Shoaf et al. [29] developed a load capacity limits model for manual lowering, pushing, pulling and carrying activities. In another study, Kai Way Li and Chih Fung Liu [30] determined the maximum acceptable weight of handling in combined MMH tasks. Iridiastadi [31] found that MAWL for combined MMH was significantly lower than MAWL previously published tested based on a single activity [32].

Combined activities are prevalent in industry, such as loading and unloading material from pallet to machine or conveyor. More researches are needed in this area due to the conflicting results combined MMH has exhibited in comparison to individual MMH activity in focused research.

In many industrial situations, the key question is not how much weight per lift a worker can lift but how frequently the worker is capable of lifting the given weight safely without straining or overexerting himself. According to Mital et al. [33] and Fox and Smith [34], the frequency of handling is the most critical task characteristic that influences an individual’s capability in performing MMH activities. Hence, frequency is a significant characteristics that influences an operator’s capability to perform lifting tasks [35]. Widia and Dawal [36] also reported a significant association between frequency and awkward body posture. The results showed that the majority of MMH workers (82.5%) experienced MSD symptoms. According to Marras et al. [37], the nature of physical work in the manufacturing industry has evolved towards low force and highly repetitive tasks in order to achieve productivity targets. Several studies have reported that work productivity is correlated to WRMSDs [38–40], which may be the consequence of highly repetitive tasks that lead to higher risk of contracting WRMSDs.

At present, there is scarcity of studies in the literature in which frequency is used as the quantifying variable for combined MMH tasks, particularly with regards to lifting light loads. Among the notable works in this area are those reported by Snook and Irvine [41] and Fox and Smith [34]. In the former study, a heavy load tote box was used to determine the maximum acceptable frequency limit (MAFL) in a symmetric lifting task, whereas the latter study use a light load box for the same purpose. Nonetheless, none has focused on determining the MAFL for combined MMH tasks such as lifting, carrying and lowering loads.

Most studies related to this subject had been carried out in the US and Europe. Hence, the data presented in these studies cater to Occidental populations [19, 42]. Wu [7] found that the Chinese population, in general, has smaller body sizes and lifting capacities, when compared to those in the US and Europe population. In recent years, researchers have looked into the effect of task demands on physiological or psychophysical experience among the Malaysian population [43, 44]. Nevertheless, it seemed that, the lifting capacities of the Malaysian population have been omitted in these studies, in particular, the MAFL. As mentioned previously, the number of industrial accidents and the cases of WRMSDs in Malaysia have escalated at annual rate despite the availability of MMH guidelines. More importantly, the individual lifting capacity amidst Malaysian males must be identified in order to minimise risk of male workers towards MSDs, apart from enhancing their productivity. For these reasons, it is deemed crucial to determine the MAFL amongst the Malaysian population. With this in mind, this study determined the MAFL and analysed the impact of lifting loads on energy expenditure and rating of perceived exertion (RPE) for combined MMH tasks performed by Malaysian males.

## Methods

### Subjects

Ten healthy Malaysian males without historical records of MSD were recruited for this study. A pilot study was conducted with three male subjects to test and to refine the proposed methods and procedure prior to the main study. The subjects were selected based on their age, gender and state of health. Initially, the subjects were given briefing regarding the purpose of the study and they were requested to sign a written consent form as evidence that they fully agreed to participate in the experiment. The subjects were introduced to a practical session prior to the experiment so that they could familiarise themselves with the experimental procedure. Approval was obtained for the methodology used in this study from the University of Malaya Research Ethics Committee (UMREC) (Ref: UM.TNC2/RCH/UMREC). Table 1 summarises the mean and standard deviation values of the anthropometric dimensions (height, knuckle height, waist height and sternum height).

**Table 1:** Anthropometric and isometric strength measurements of the Malaysian male subjects.

Variable	Mean	SD	Range
Age (years)	27.50	4.86	22–36
Weight (kg)	69.60	17.06	50–105
Height (cm)	167.10	7.56	150–176
Knuckle height (cm)	72.20	3.85	68–80
Waist height (cm)	95.50	4.65	86–102
Elbow height (cm)	104.50	5.19	96–109
Shoulder height (cm)	135.80	5.92	126–144
Sternum height (cm)	126.90	6.71	114–135
Waist circumference (cm)	97.35	13.89	83–105.5
Bicep circumference (cm)	31.53	4.70	24–39
Forearm circumference (cm)	26.14	2.61	22–30
Shoulder circumference (cm)	107.35	8.18	98–126
*Isometric strength (N)*			
Arm	229.17	69.34	142.33–363.23
Back	479.81	169.29	215–662.93
Shoulder	390.53	118.08	169.03–520.43
Leg	499.65	143.10	290.6–757.63

(*N =* 10)

### Experimental design

The experimental task was designed based on loading and unloading tasks observed in the industry as well as those reported in past studies [45, 46]. Based on the field observations, the lifting height was kept constant between 73 and 130 cm (table height), which is approximately equaivalent to the average knuckle and sternum height. The variables and conditions of the experiment are summarised in Table 2.

**Table 2:** Variables and conditions of the experiment.

	Variable	Levels or conditions
Independent variable	Load	1 kg and 5 kg
	Lifting height	73–130 cm and 130–73 cm
	Pace	Metronome-paced (slow, high initial frequency) and unpaced
Dependent variable	Maximum acceptable frequency limit (MAFL) (cycles/min)	
	Energy expenditure (kcal/min)	
	Rating perceived exertion (RPE)	
Controlled variable	Simulated automotive parts (Fig 1)	1 kg = car body interior (81 cm × 15.5 cm × 2.5 cm) 5 kg = bumper (125 cm × 22 cm × 5 cm)
	Task duration	45 minutes
	Temperature	23°C

The subjects were requested to wear comfortable clothing for the experiment. Energy expenditure and RPE were recorded as the response variables when the subjects performed the task. Fig ‎1 illustrates the experimental set up applied in this study.

**Fig 1:**
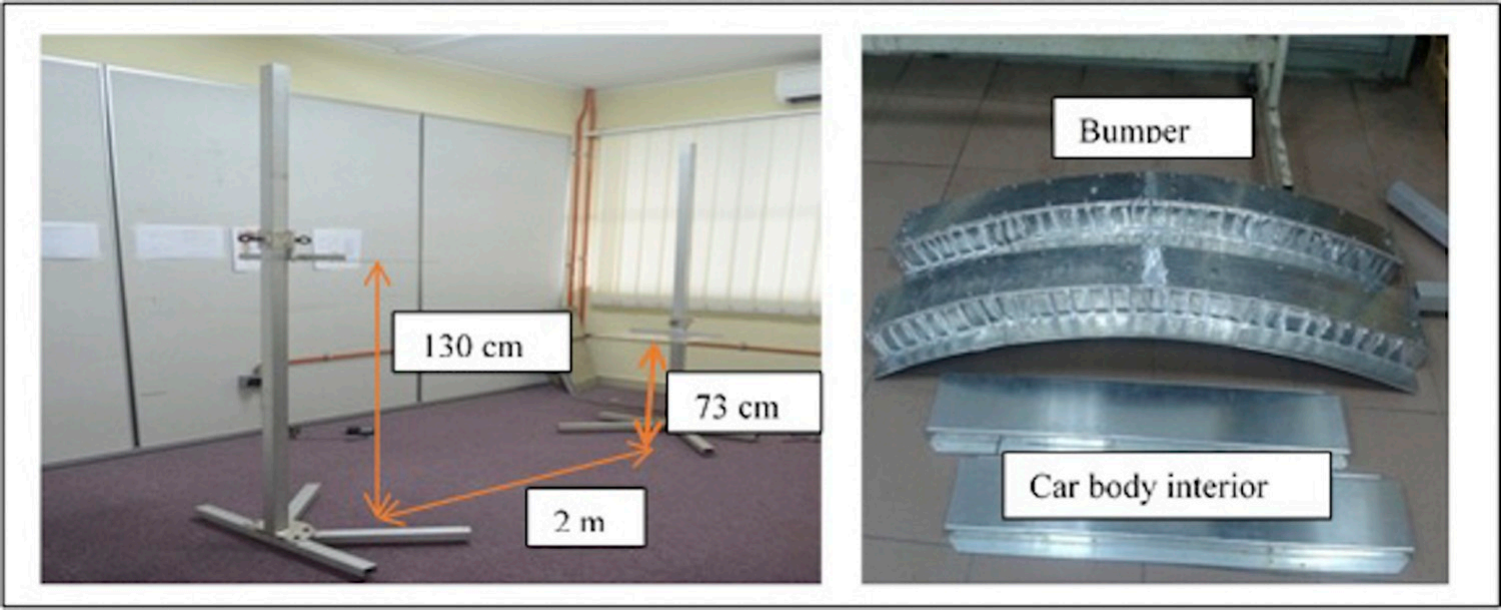
Experimental set up.

### Task

**Fig 2:**
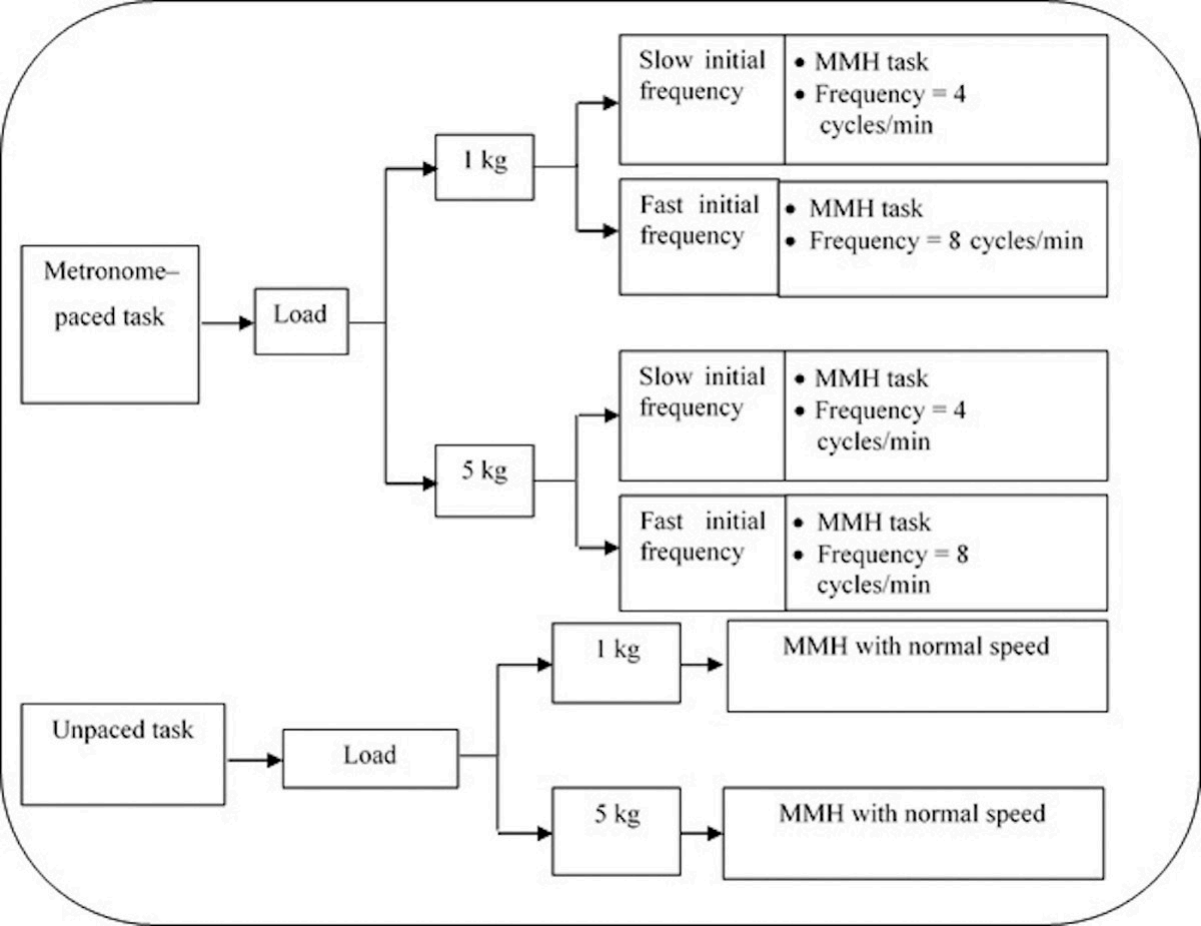
Task conditions.

The combined MMH task for one cycle time is defined as follows: the subject is required to lift the part from a specific height to his elbow height, carry the part against his waist for a distance of 2 m, place the part from his elbow height to a specific table height, and then, walk back towards the starting position while carrying the same part. As for data collection, each subject was required to perform each of the six task conditions over a period of 45 minutes in a random sequence. Each subject was encouraged to lift in any body posture that he found to be most comfortable based on the perception towards task demand. The instructions given to the subjects were consistent with those of prior studies [7, 24, 34]. The six task conditions had been divided into two experimental sessions, as portrayed in Fig 2.

### Experimental procedure

A psychophysical approach was applied to determine the MAFL of the subjects for each lifting task. The instructions given to the subjects were similar to those used by Fox and Smith [34].

The subjects were ensured that they understood the instructions and what was expected of them. The subjects were informed to work as hard as they could without straining themselves or becoming tired, weak and overheated [34, 41]. The subjects were asked to lift the given load using a customised metronome beeper. The experimental procedure for each session is described briefly as follows:

#### Session 1: Metronome-paced task

In this session, each subject was required to adjust his frequency by calling out changes to the experimentalist who then adjusted the metronome. The subject would call out ‘increase’ to increase the frequency and likewise, he would call out ‘decrease’ to decrease the frequency. The experimentalist adjusted the frequency in steps of two cycles/min response according to the command given by the subject. If the subject required only slight adjustment, the frequency was adjusted by one cycle/min. It is highlighted here that the subjects were unaware of the actual frequency. It had been perceived that calling out for adjustments of the frequency to the experimentalist enabled each subject to perform the task at a steady pace without disrupting his work rhythm. This reflected as an effective procedure as it would have been undesirable if the subject had to pause his work merely to manually adjust the frequency himself by pressing the button on the metronome beeper. The subjects were allowed to adjust their frequency for the initial 40 minutes out of the 45-minute experimental session. This frequency adjustment period had been selected based on that suggested by Snook and Irvine [41] and Fox and Smith [34].

#### Session 2: Unpaced task

Two additional sessions were incorporated into the experiment for 1 and 5 kg load without the use of metronome beeper in order to provide a pace cue during these extra sessions. Similar to the previous procedure, the subjects were required to work as hard as they could for 45 minutes at a constant pace (as much as they could possibly manage) without the metronome beeper. The experimentalist counted the number of cycles per minute performed by each subject for every 5 minutes throughout the 45-minute experimental session.

The primary purpose for this unpaced task sessions was to simulate a typical work session in the industry. Unlike the metronome-paced session, these sessions reflect ‘internally-paced’ sessions since they are dependent on the subject’s own internal assessment of pace.

The aim of the study is to determine the difference in pace with respect to the metronome-paced session and how consistent the subjects were in maintaining their pace without external interference.

### Equipment

An Actiheart monitoring device was used in this study to determine energy expenditure, which was derived from heart rate and activity data. These recorded data for energy expenditure and heart rate were downloaded into a computer for further analysis. The Actiheart is a valid method that can be used to measure energy expenditure while performing work [47,48]. The data of energy expenditure were averaged for each experimental task and were analysed using SPSS software. The Actiheart monitoring device, which was worn on the chest, has two electrodes connected by a short lead cable and clipped onto two standard ECG pads. The device is comfortable to wear for ambulatory activity and heart rate recording since it is a self-contained instrument.

### Statistical analysis

A two-way ANOVA for repeated measures was carried out to determine the variances in MAFL, energy expenditure and RPE between the experimental sessions (paced and unpaced tasks). Statistical comparisons of MAFL for both paced and unpaced tasks were performed via paired sample t-test. Cohen (1988) proposed the following interpretation of d values (paired sample t-test). A d value close to 0.2 is a small effect, while d near 0.5 reflects a medium effect, and d near 0.8 refers to a large effect. A confidence interval of 95% was applied in this analysis. The principle behind significance value is that the p value should be less than 0.05 (CI 95%). Unless otherwise specified, the data are presented as mean±standard deviation.

## Results

### Metronome-paced and unpaced task sessions

**Fig 3:**
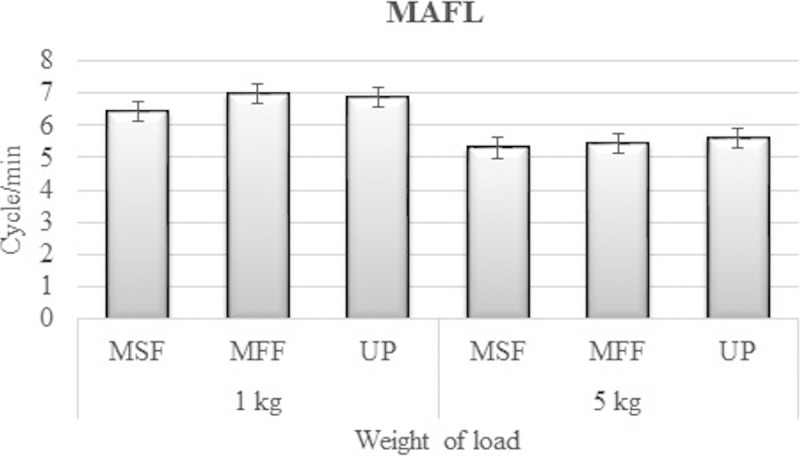
The mean and standard error of MAFL based on the lifting load (MSF = Metronome Task-slow initial frequency; MFF = Metronome Task-fast initial frequency; UP = unpaced task).

**Fig 4:**
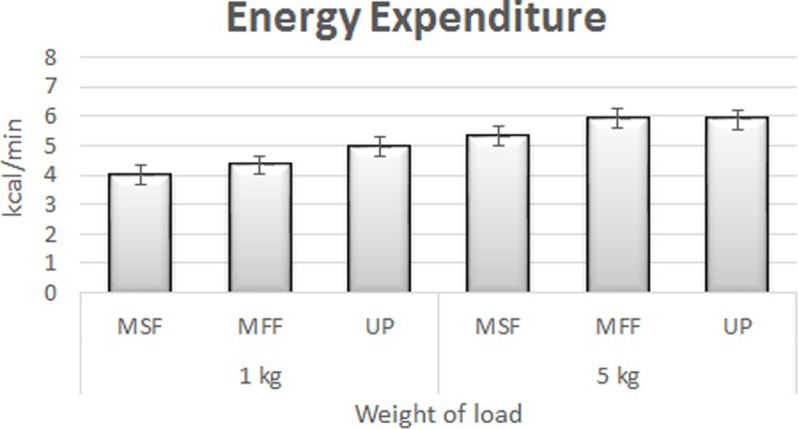
The mean and standard error of energy expenditure based on the lifting load (MSF = Metronome Task-slow initial frequency; MFF = Metronome Task-fast initial frequency; UP = unpaced task).

The mean values for MAFL and energy expenditure are summarised in Figs 3 and 4, respectively. The MAFL seemed to decrease with increment in lifting load during the six sessions. On the contrary, the energy expenditure increased when the lifting load was increased. The MAFL for 5 kg load (5.38±0.69 metronome; 5.62±0.83 unpaced) was significantly lower than that for 1 kg load (6.73±0.71 metronome; 6.86±0.84 unpaced), with a difference of 22.3% and 19.87% during metronome-paced and unpaced tasks, respectively. The two-way ANOVA outputs revealed that load had a statistically significant effect on both MAFL and energy expenditure during metronome-paced and unpaced tasks (*p* < 0.01). Based on the two-way repeated measures of ANOVA with Greenhouse-Geisser correction, it was found that the mean value for MALF differed significantly between the loads (F(1, 86) = 9.2, p < 0.000, partial η2 = 0.1). Meanwhile, the paces (F(1, 86) = 3.01, p > 0.000, partial η2 = 0.04) and the interactions between loads and paces (F(1, 86) = 0.09, p > 0.000, partial η2 = 0.00) displayed insignificant variances in MALF. The outcomes showed that the differences in energy expenditure between 1 kg (4.16±0.95 metronome; 4.98±0.84 unpaced) and 5 kg (5.62±0.74 metronome; 5.88±0.45 unpaced) for metronome-paced and unpaced tasks were 29.86 and 16.57%, respectively.

The ANOVA results revealed that the lifting load had a significant effect on the overall RPE (*p* < 0.01). When the lifting load was increased from 1 to 5 kg, the overall RPE increased slightly from 1.4 to 2.4, *i*.*e*. from ‘very weak’ to ‘weak’. Table 3 summarises the ANOVA results.

**Table 3:** Analysis of variance for the maximum acceptable frequency limit, energy expenditure and rating of perceived exertion.

	MAFL				Energy Expenditure				RPE			
Source	*df*	F	*p*	*η*_*p*_^*2*^	*df*	F	*p*	*η*_*p*_^*2*^	*df*	F	*p*	*η*_*p*_^*2*^
**Load**	1	9.2	0.00*	0.10	1	9.23	0.00*	0.08	1	11.5	0.00*	0.17
**Lifting pace**	1	3.01	0.08	0.04	1	3.01	0.08	0.00	1	0.48	0.49	0.01
Load*Lifting pace	1	0.09	0.77	0.00	1	0.09	0.77	0.00	1	0.90	0.35	0.02

* *p<0*.*01*

Table 4 presents no significant difference (*p* > 0.05) in the frequency between paced and unpaced measurements for both lifting loads. This indicates that the subjects were indeed consistent in maintaining their work pace throughout the experimental sessions.

**Table 4:** Paired *t*-test results for paced and unpaced tasks.

	Paced		Unpaced			95% CI for Mean Difference			
Outcome	M	SD	M	SD	n		R	t	df
**Frequency 1 Kg**	6.45	0.76	6.86	0.84	10	-0.99, 0.18	0.15	-1.56	9
**Frequency 5 Kg**	5.3	0.79	5.62	0.83	10	-1.03, 0.4	0.34	-1.01	9

### Rating of perceived exertion (RPE)

Table 5 lists the RPE values recorded while the subjects’ were lifting load at the end of each lifting task. The perceived exertion for forearm, upper arm, lower back, shoulder and the entire body increased with increment in lifting load. The maximum increase in the perceived exertion was observed for forearm and lower back among all the other body parts investigated in this study. The minimum increase in the perceived exertion was noted the shoulders.

**Table 5:** The mean and standard deviation of RPE for the Malaysian male subjects during the metronome-paced and unpaced task sessions.

RPE	Metronome-paced Task		Unpaced Task	
	1 kg	5 kg	1 kg	5 kg
**Forearm (R)**	1.38±1.10	2.55±1.58	1.45±1.01	3.25±1.84
**Forearm (L)**	1.48±1.11	2.75±1.64	1.45±1.01	3.25±1.59
**Upper arm (R)**	1.28±0.87	2.25±1.41	1.20±0.79	2.85±1.67
**Upper arm (L)**	1.63±1.14	2.55±1.72	1.35±1.00	3.15±1.63
**Shoulder (R)**	1.10±1.02	1.78±1.52	1.05±0.89	2.15±1.53
**Shoulder (L)**	1.15±1.23	2.00±1.96	0.95±0.96	2.25±1.99
**Lower back (R)**	1.58±1.31	2.48±2.07	1.20±1.03	2.55±1.89
**Lower back (L)**	1.60±1.42	2.53±2.11	1.20±1.03	2.80±2.15
**Entire Body**	1.48±1.01	2.43±1.38	1.30±0.82	3.05±1.67

## Discussion

The highlight of this study is the MAFL for 1 and 5 kg loads. Based on the outcomes retrieved, the MAFL appeared to decrease with increase in lifting load, while both energy expenditure and RPE increased when the lifting load was increased. The mean values for lifting frequencies were 6.8 and 5.5 cycles/min for 1 and 5 kg load, respectively. The mean value of MALF decreased by 19.11% from the load weight of 1 to 5 kg, which is in agreement with the findings reported by Fox and Smith [34] that attained a near similar percentage decrease of 24.7%. Nevertheless, the mean value of MALF for this present study was significantly lower than those observed in another psychophysical research related to MMH tasks [34]. This is attributable to the differences in the type of task. This present study involved the combination of MMH tasks (lifting, carrying and lowering), while past studies only incorporated individual tasks, such as symmetric or asymmetric lifting tasks. The results could also reflect the variations in the physiques (anthropometric dimensions and isometric strengths) of different populations. The percentage difference in the arm strength of the subjects in this study was 57.34% lower than that reported in a previous study [49]. In general, the Asian population has a smaller average body size and also relatively weaker strength in their upper extremities, when compared to the Occidental populations [6, 7]. Therefore, it is perfectly understandable if the mean value for MALF recorded in this study significantly differed, in comparison to those reported in prior studies. Hence, the recommended maximum work pace is 2400 and 3360 cycles/8 hours for 1 and 5 kg load, respectively. Based on a survey in a similar work task within the context of two Malaysian automotive industries [36], the recommended work pace is bound to increase work productivity by 52.30 and 66.67% for 1 and 5 kg load, respectively. In other industries, the work pace should be reduced by about 20% to prevent the worker from succumbing to WMSDs. It has been perceived that it is critical for organisations to consider the recommended maximum work pace in their work design and work productivity planning to hinder cases of muscle fatigue and to reduce the risk of WRMSDs among workers.

The increase in energy expenditure also displayed agreement with [50, 51] upon increase in lifting load. The mean values for energy expenditure were 4.16 and 5.62 kcal/min for 1 and 5 kg load, respectively. The values are below the maximum limit of 7.125 kcal/min for standing arm lifting task based on the guidelines provided by NIOSH [15]. This signifies that the task given to the subjects was not stressful, but was well within their lifting capacity. From a logical stance, the 5 kg load would have produced more biomechanical strains in the upper extremities, when compared to that exerted by 1 kg load. Additionally, different limiting factors were noted for these two loads based on the disparity in the percentage of aerobic capacity [34]. The 1 kg lifting task may have been limited more by the mechanics of the task (*i*.*e*. the speed of movement), in comparison to the metabolic and fatigue impacts of performing the task. On the contrary, the 5 kg load may have been limited more by the metabolic and fatigue impacts of the load. The subjects worked harder with the 5 kg load, but they lifted it for fewer number of cycles per minute. These observations have important implications when designing MMH tasks.

The perceived stress levels of the subjects increased when the lifting load was increased. The variance between the 1 and 5 kg loads appeared to be significant in many aspects. In general, the 5 kg load resulted in higher exertion, when compared to that for 1 kg load in the right forearm (85%), left forearm (86%), right upper arm (76%), left upper arm (57%), right shoulder (61%), left shoulder (74%), right lower back (57%) and the entire body (64%). However, the average RPE for the 5 kg load was close to ‘scale 3’ (moderate). This finding is in good agreement with the levels of perceived exertion reported in a similar study involving MMH tasks [34]. This indicates that the subjects perceived that the given task was not really stressful. In precise, the subjects performed the combined MMH task as quickly as possible without exhausting themselves or becoming overheated. Fox and Smith [34] found that the body parts with the highest stress levels for symmetric lifting task were the lower back and legs. Turning to this study, the body parts with the highest stress levels were the forearms and lower back, which are in line with that reported by Wu [7]. This displays the differences in the perceived response between individual lifting tasks and combined MMH tasks.

## Limitations of the study

One of the main limitations in this study is the relatively small number of subjects involved and therefore, the characteristics are not representative of the whole Malaysian population. All the subjects are males and they do not have any experience in MMH tasks. Hence, it is important to further explored the MALF amongst experienced workers [52]. With that, it is recommended that a larger sample size should be used in future studies by including females and subjects from other age groups. It is more desirable if the subjects are recruited from the industry, specifically those involved directly in MMH tasks. This is particularly important in order to obtain a holistic view on the crucial parameters in MMH tasks where in these parameters can be used to design MMH tasks by incorporating safety aspects. Beside, only energy expenditure measurements were considered in this study, despite other available physiological measurements, such as muscle activity, which are equally significant. Hence, this study proposes that other approaches (*e*.*g*. EMG, motion study) should be embedded to record muscle activity and joint moments in future studies. Recording the postures of the subjects and, taking into account muscles such as erector spinae, biceps brachii and flexor carpi radialis, are bound to offer more accurate estimation of the overall muscular loadings. Such information will be useful to help boost the productivity of workers involved in MMH tasks, apart from minimising the risk of WRMSDs.

## Conclusion

This study had determined the MAFL for combined MMH tasks amongst selected Malaysian males. Two loads were considered in this study: 1 kg and 5 kg. The study outcomes portrayed that the lifting load exerted a significant effect on the MAFL, energy expenditure and RPE. The mean of MALF decreased by 19.11% from the load weight of 1 to 5 kg. The presented findings have important implications in industrial practice since they can be used as the groundwork to design MMH tasks by weighing in the aspects of MAFL and energy expenditure amidst the workers.

## Supporting information

S1 File Raw data file. (XLSX)
